# Fast and Accurate Multiplex Identification and Quantification of Seven Genetically Modified Soybean Lines Using Six-Color Digital PCR

**DOI:** 10.3390/foods12224156

**Published:** 2023-11-17

**Authors:** Alexandra Bogožalec Košir, Sabine Muller, Jana Žel, Mojca Milavec, Allison C. Mallory, David Dobnik

**Affiliations:** 1 Department of Biotechnology and Systems Biology, National Institute of Biology, Večna pot 121, 1000 Ljubljana, Slovenia; 2 Stilla Technologies, Biopark 1, Mail du Professeur Georges Mathé, 94800 Villejuif, France

**Keywords:** digital PCR, quantification, multiplexing, genetically modified organisms, 6-color system

## Abstract

The proliferation of genetically modified organisms (GMOs) presents challenges to GMO testing laboratories and policymakers. Traditional methods, like quantitative real-time PCR (qPCR), face limitations in quantifying the increasing number of GMOs in a single sample. Digital PCR (dPCR), specifically multiplexing, offers a solution by enabling simultaneous quantification of multiple GMO targets. This study explores the use of the Naica six-color Crystal dPCR platform for quantifying five GM soybean lines within a single six-plex assay. Two four-color assays were also developed for added flexibility. These assays demonstrated high specificity, sensitivity (limit of detection or LOD < 25 copies per reaction) and precision (bias to an estimated copy number concentration <15%). Additionally, two approaches for the optimization of data analysis were implemented. By applying a limit-of-blank (LOB) correction, the limit of quantification (LOQ) and LOD could be more precisely determined. Pooling of reactions additionally lowered the LOD, with a two- to eight-fold increase in sensitivity. Real-life samples from routine testing were used to confirm the assays’ applicability for quantifying GM soybean lines in complex samples. This study showcases the potential of the six-color Crystal dPCR platform to revolutionize GMO testing, facilitating comprehensive analysis of GMOs in complex samples.

## 1. Introduction

The rise in the number and diversity of genetically modified organisms (GMOs) on the global market presents a challenge for GMO testing laboratories as well as policy makers. Today, the most commonly used approach in GMO testing laboratories is to first screen the sample for the presence of GMOs by targeting the most commonly present GM elements with quantitative real-time PCR (qPCR). When or if the screening indicates the occurrence of one or more GM lines, the presence of specific individual GM lines must be confirmed, and the specific line quantified. Although until now qPCR was the undisputed gold standard in GMO testing, the rising number of GMOs requiring quantification in a single sample makes the analysis by qPCR progressively more costly. In addition, the rise in GMO product complexity renders individual GM line quantification more challenging. This has encouraged researchers to look for other options and develop novel approaches. One approach to more efficient GMO testing is digital PCR (dPCR). Due to its binary nature, dPCR provides absolute quantification without the need of a standard curve, which is advantageous especially in the fields where (certified) reference materials (CRMs) used for standard curves are lacking. Despite the dPCR’s limited dynamic range, which is restricted by the number of partitions [1,2], the precision of quantification is generally higher than that of qPCR [3,4]. However, perhaps the biggest advantage for GMO testing is robust multiplex quantification. This is particularly important for complex samples where qPCR-based quantification is difficult due to sensitivity to inhibitors often present in GMO samples [5,6,7,8,9,10].

Over the past few years, several assays for GMO quantification have been transferred from qPCR to dPCR. Although the majority were simplex or duplex assays [11,12,13,14,15,16,17], a handful of studies have regrouped simplex assays in a single channel to attain an overall higher level of multiplexing [18,19,20,21,22,23]. Indeed, in the past, the feasibility of routine multiplex quantification with dPCR has been hampered by the limitations of the platforms, either due to a limited number of detection channels or to a low partition number. Thus, the highest multiplexing dPCR assays have been limited to the simultaneous quantification of several GMOs in a single channel, precluding the possibility of distinguishing different GMOs from one another. Although the European Union legislation allows for such grouped quantification [24], many national authorities must identify the individual GM lines present in samples for official control and monitoring in the process of identifying the presence of GMOs that are no longer authorized and GMOs pending authorization. By using different probe concentrations detected in the same color channel, reasonable levels of individual multiplexing have been attained [19,22]. However, such a tedious approach is far too complex for routine use in a GMO testing laboratory. To increase the number of targets that can be individually quantified in a single dPCR assay and thus circumvent such detection complexities, platforms with a higher number of channels are being developed.

Stilla Technologies’ 6-color Crystal Digital PCR platform was previously used for multiplex quantification in cancer research and diagnostics, including for the quantification of 19 of the most prevalent *EGFR* sensitizing and resistance mutations in non-small cell lung cancer plasma samples [25,26]. To test the utility of the Naica 6-color Crystal Digital PCR system in the field of GMO testing, where the quantification of a low target concentration in a complex sample is needed, we assessed its robustness to quantify five GM soybean lines (CV127, DP305324, MON87701, MON87708, MON87769) and the soybean reference gene lectin (*Le1*; Supplementary Table S1). The major advantage of this setup is, as opposed to the grouped quantification performed in the previously described assay [20], that the 6-color system enables the identification and quantification of all five individual lines in a single 6-plex assay. To demonstrate the flexibility of dPCR in general and the Naica 6-color system in particular, we added two additional GM lines known commercially as “RoundUp ready” and “RoundUp ready II” (MON40-3-2 and MON89788, respectively) to the panel and divided it into two 4-color assays (4-plex I and II; Supplementary Table S1).

The performance of all three assays (6-color assay—6-plex assay, 4-color assays—4-plex assays I and II) was tested for specificity, sensitivity, repeatability and fitness for purpose. The results highlight the platform’s ability to offer enhanced specificity, sensitivity and precision. This work emphasizes the potential of the 6-color crystal dPCR platform to reshape GMO testing methodologies, enabling more effective and comprehensive analysis of GMOs in complex samples.

## 2. Materials and Methods

The “Minimum Information for Publication of Quantitative Digital PCR Experiments for 2020” (dMIQE2020) checklist [27] was followed and is given at the end of the Supplementary Materials file.

### 2.1. Test Materials

Test material was divided into two sets, nonblinded and blinded samples. Nonblinded samples were used for characterization of the assays (sensitivity, repeatability testing) and blinded samples were used to determine fitness for purpose and in vitro specificity, for these samples the content of the specific GM lines was unknown to the operator.

#### 2.1.1. Nonblinded Samples

Two DNA mixes, corresponding either to the five GM soybean lines targeted by the 6-plex assay or to the seven lines targeted by the combined 4-plex assays, were prepared from the DNA extracted from the following genetically modified (GM) soybean certified reference materials (CRMs): AOCS 0311-A, AOCS 0911-C, AOCS 0809-B, AOCS 0809-A, AOCS 0906-B (American Oil Chemists’ Society, S. Boulder, Urbana, IL, USA), ERM-BF-426d and ERM-BF410g (Directorate F—Health and Food, European Commission, Directorate General, Joint Research Centre, Geel, Belgium). They contained 99.05% (mass/mass) MON87708, 96.32% (mass/mass) CV127, 99.94% (mass/mass) MON87769, 99.94% (mass/mass) MON87701, 100% (mass/mass) MON89788, 10% (mass/mass) DP305423 and 10% (mass/mass) MON40-3-2. The copy number of individual targets in the DNA mixes was assessed using Bio-Rad’s QX100 platform for dPCR. In brief, each of the two DNA mixes were diluted 50× and tested in duplicate using simplex assays targeting corresponding GM targets and the reference gene *Le1*. All reactions (total volume, 20 μL) contained 10 μL ddPCR Supermix for probes (no dUTP; Cat. No. 1863024; BioRad, Pleasanton, CA, USA), 6 μL primers and probe mix (final concentration in Supplementary Table S2). For the droplet generation, droplet generator cartridges (DG8; BioRad) were combined with the droplet digital system (QX100; BioRad). The droplets generated were transferred to 96-well plates, and the PCR reactions were carried out using a thermal cycler (C1000 or T100; BioRad, USA) under the following amplification conditions: 10 min DNA polymerase activation at 95 °C; followed by 40 cycles of a two-step thermal profile of 30 s at 94 °C for denaturation and 60 s at 60 °C for annealing and extension; followed by 10 min at 98 °C; and then cooling to 4 °C. After the thermal cycling, the 96-well plates were transferred to a droplet reader (QX100/QX200; BioRad), and the data were gathered. The data were analyzed using the software package provided with the dPCR system (QuantaSoft 1.7.4.0917; BioRad) and Microsoft Excel, where a partition volume of 0.715 nL [28] was used to calculate copy number, which was further used to calculate GM%. A dilution series was performed, and each dilution was tested in duplicate to determine the copy number of *Le1*. Based on the *Le1* copy number and the GM%, the copy number per reaction (25 µL reaction containing 5 µL DNA) of all targets was assigned (Supplementary Tables S3 and S4).

#### 2.1.2. Blinded Samples

Four samples (samples A–D) from routine studies were chosen as test materials in the fitness-for-purpose study (Supplementary Table S5). The four samples were analyzed using qPCR in a routine analysis. In brief, the samples were first tested for the presence of soybean with a simplex assay targeting *Le1* in three concentrations, each in two technical repeats. Detection of specific GM soybean lines followed using a so called pre-spotted plate, with witch the presence or absence of specific in the EU authorized GM soybean lines was tested in two technical repeats. All reactions (total volume, 10 μL) were comprised of 5 μL 2× Universal Master Mix (Applied Biosystems), 3 μL primers and probe (final concentration in Supplementary Table S2) and 2 μL DNA. Reaction was performed either on ABI7900HT Fast or ViiA7 qPCR platform (Applied Biosystems). Additionally, two control samples, one containing 17 soybean lines (sample E) and one containing 23 maize lines (sample F) (Supplementary Table S6), were used for determining in vitro specificity.

### 2.2. DNA Extraction and Purification

DNA was extracted and purified in one extraction parallel either by the cetyltrimethyl-ammonium bromide (CTAB) protocol, with RNase-A and proteinase-K for removal of RNA and protein, respectively, as described in Annex A.3 of ISO21570:2005 [29] or by NucleoSpin Food kit (Macherey-Nagel GmbH & Co. KG, Düren, Germany). CTAB was used for samples A and B (10 g of starting material) and for all certified reference materials (2 g of starting material for reference material purchased at AOCS and 200 mg for material purchased from Directorate F, JRC EC), while NucleoSpin Food kit (200 mg of starting material) was used for samples C and D. DNA was resuspended in 150 µL of water in the case of CTAB extraction or eluted twice with 100 µL elution buffer for NucleoSpin Food kit. Samples C and D extracted with NucleoSpin Food kit were additionally added 300 µL of nuclease-free water. All extractions included a negative extraction control, where nuclease-free water was used in place of sample.

Dilutions of the extracted stock DNA solutions were supplemented with 2 µg/mL sheared salmon sperm DNA (Invitrogen by Thermo Fisher Scientific, Waltham, MA, USA) in nuclease-free and protease-free water (Sigma-Aldrich Chemie GmbH, Munich, Germany). All DNA extracts and samples were stored below −15 °C until further use.

### 2.3. In Silico Specificity Prediction

The potential interaction between primers and probes and in silico multiplex specificity for the targets in the 6-plex assay were evaluated as previously described [20]. For both 4-plex assays, all primers and probes were evaluated for potential intra- and intermolecular interactions using Autodimer [30], and the multiplex specificity was assessed using Primer-BLAST [31]. Standard parameters were used, with the exception of database, where nr database was used instead of RefSeq mRNA and search against the complete database (no specific organism was selected). As the specificity of the six assays present in both 6-plex and 4-plex assays was already assessed [20], we used Primer-BLAST to interrogate the potential cross-reactivity of the new combinations created in the two 4-plex assays of MON40-3-2 with DP305423, MON87708 and MON87701 and of MON89788 with CV127, MON87769 and *Le1*. Only amplicons that arose from two different oligonucleotides were considered as cross-reactivity.

### 2.4. Primers, Probes and PCR Methods

The primer and probe sequences were taken from GMOMETHODS (http://gmo-crl.jrc.ec.europa.eu/gmomethods/, accessed on 12 May 2023), with the entry names of QT-TAX-GM-002 for soybean reference gene lectin (*Le1*) and QT-EVE-GM-012 for MON87708, QT-EVE-GM-011 for CV127, QT-EVE-GM-002 for MON87769, QT-EVE-GM-010 for MON87701, QT-EVE-GM-006 for MON89788, QT-EVE-GM-008 for DP305423 and QT-EVE-GM-005 for MON40-3-2. The assay setup for each 6-plex and 4-plex assay and the final concentrations of all primers and probes can be found in Supplementary Tables S1 and S2. Primers and probes were purchased from Eurofins MWG Operon (Ebersberg, Germany), Integrated DNA Technologies (Leuven, Belgium) or Eurogentec (Liege, Belgium), shipped lyophilized and diluted in nuclease-free and protease-free water (Sigma-Aldrich Chemie GmbH, Munich, Germany) or shipped as 100 mM suspensions.

### 2.5. Multiplex Crystal™ Digital PCR Conditions and Imaging

The 4-plex assays were performed in 25 µL reactions containing 1x PerfeCTa qPCR ToughMix UNG (Quanta Biosciences, Gaithersburg, MD, USA), 100 nM fluorescein disodium salt, high purity (VWR, Fontenay-sous-Bois, France), 5 µL DNA sample and a mix of either 4-plex I or 4-plex II primers and probes at the indicated concentrations (Supplementary Table S4). The 6-plex assays were performed in the same way as the 4-plex assays but using 1x PerfeCTa Multiplex qPCR ToughMix and a mix of 6-plex primers and probes.

Microfluidic Sapphire chips, composed of four chambers, were loaded with 25 µL PCR mixes, placed in the Naica Geode, a pressurized thermocycler, to generate stable 2D arrays of monodispersed droplets, called droplet crystals, followed by thermocycling. Thermocycling conditions were as follows: 95 °C for 3 min, followed by 50 cycles (4-plex assays) or 45 cycles (6-plex assays) of 95 °C for 10 s and 60 °C for 15 s. Upon PCR completion, the stable droplet crystals were imaged with a 6-color prototype version of the Naica™ 6-color System (Stilla Technologies, Villejuif, France, [25]; Supplementary Figure S1). Once acquired, images generated by the 6-color prototype reader were converted to compatible files and analyzed using prototype Crystal Miner analysis software extended to six dimensions for automated data extraction and quantification. The thresholds were set manually at amplitudes of approximately 4400 (FAM), 9000 (YY), 7000 (Cy3), 11,000 (ROX), 17,000 (Cy5) and 7000 (Cy5.5) relative fluorescence units (RFU) for the 6-plex assay, at 6500 (FAM), 7000 (HEX), 15,000 (Cy5) and 7500 (Cy5.5) for 4-plex I and at 5000 (FAM), 8000 (HEX), 12,000 (ROX) and 6500 (Cy5.5) for 4-plex II. The data were rejected from subsequent analysis if a low number of droplets (<15,000) was measured per 25 µL reaction.

### 2.6. Multiplex Crystal™ Digital PCR Chip Setup

Two different serial dilutions (nonblinded samples) composed of either 11 or 7 dilution points (Supplementary Tables S3 and S4) of a mix of GM soybean DNA were assessed with the 4-plex and 6-plex assays, respectively. The chip setup of both 4-plex assays and the 6-plex assay was performed as indicated in Supplementary Figures S2 and S3. In short, each dilution point was tested in eight technical repeats in two different days, four repeats in each day. No template controls (NTCs) were run in at least two technical repeats per assay.

A mixed set of four real-life GM soybean samples (samples A–D) and control samples (samples E and F) with known GM composition was analyzed in a blinded manner. Each sample was tested in duplicate for all three assays. The chip setup of both 4-plex assays and the 6-plex assay was performed as indicated in Supplementary Figures S2 and S3.

### 2.7. Multiplex Crystal™ Digital PCR Data Analysis

#### 2.7.1. Color Compensation

In order to compensate for fluorescence spillover between the different channels and ensure the correct representation of background in all channels, monocolor controls for each panel were performed as follows: from a pool of DNA containing all GM lines represented in each panel, each GM line was amplified by adding uniquely the primers and the probe corresponding to the GM line in addition to all other probes but excluding their corresponding primers. In addition to the corresponding NTCs, all fluorescence results from the monocolor controls were processed with prototype Crystal Miner analysis software extended to six dimensions and compensation matrixes were generated as described in [26].

#### 2.7.2. Limit- of- Blank Correction Using Bayesian Method

Limit-of-blank statistical tool available at the Gene Pi online learning platform (https://www.gene-pi.com/statistical-tools/loblod-ep17a2/, accessed on 15 September 2023) was used for limit of blank (LOB) calculation. The method applied is based on a Bayesian approach adapted for dPCR. When the LOB of a target nucleic acid is nonzero, the estimated quantity of target in a test sample should be corrected by “subtracting the false positives” weighted by their probability distribution. Here, the estimation of the probability distribution of the false positives was calculated based on the results obtained for 22 and 50 negative controls corresponding to 6-plex and for 4-plex assays, respectively. According to the Bayes Rule, the probability of having *p* true positive partitions in a test sample knowing the probability *Λ* = *e − λ* that a partition is negative (where *λ* is the average number of copies per partition), follows Equation (1), where an uninformed prior distribution is used.

(1) $$P(p/\Lambda )=\sum_{k=0}^{K}P(FP=k)\;P(p/\Lambda \;,\;FP=k)\;$$

The true concentration of target nucleic acids in the well is given by Equation (2), where *v* is the average partition volume, and *Λ_true_* is the value of *Λ* which maximizes the posterior distribution *P*(*p*/*Λ*). For target detection, we consider if the posterior distribution is statistically separated from zero given a confidence level of 95%.

(2) $$Ctrue=-\frac{1}{v}\;ln(\Lambda_{true})\;$$

#### 2.7.3. Chamber Pooling

The chamber pooling was applied to a set of four and two chamber replicates of the nonblinded and blinded samples, respectively. When the test sample is loaded in more than one replicate well, then the pooling strategy allows for the detection of smaller target concentrations, and the estimated concentration (*C*) is calculated using Equation (3), where *v* is the partition volume, *M* is the number of replicate wells, and *pi* is the number of positive partitions observed among *Ni* analyzable partitions in the *i*th replicate well.

(3) $$C=-\frac{1}{v}\;ln(1-\frac{\sum_{i=1}^{M}\;pi}{\sum_{i=1}^{M}\;Ni})$$

## 3. Results and Discussion

The aim of this study was to evaluate the ability of dPCR to achieve quantification in high multiplexing conditions at a low concentration of target in complex samples. For this purpose, the two-color 6-plex assay targeting five GM soybean lines and the soybean reference gene lectin, developed by Bogožalec Košir et al. [4], was transferred to a 6-color crystal digital PCR system, generating a new 6-plex assay capable of quantifying five GM soybean lines (CV127, DP305324, MON87701, MON87708, MON87769) and soybean endogen *Le1* in one single reaction.

As there are currently at least 45 GM soybean lines present on the global market, 16 of which are multiple transformations, i.e., more than one gene was inserted in the genome of the plant (https://www.isaaa.org/gmapprovaldatabase/crop/default.asp?CropID=19, accessed on 7 November 2023), at least four additional high complexity multiplex assays would need to be designed to quantify all GM soybean lines. It was not the purpose of this study to design assays to quantify all GM soybean lines; however, with the growing number of GM lines worldwide, we recognize the need to readily add/remove targets in a multiplex system. The flexibility of the 6-color system was assessed by splitting the 6-plex assay in half and adding one GM soybean lines to each assay, creating two 4-plex assays, the first targeting DP305324, MON87701, MON87708 and MON40-3-2 and the second targeting CV127, MON87708, MON87769 and MON89788.

Although the study design did not foresee a full validation procedure, the evaluation of the assays was guided by the Definition of Minimum Performance Requirements for Analytical Methods of GMO Testing [23], a document on performance assessment and acceptance of methods for legal compliance with the European Union GMO legislation. Among other parameters such as specificity, precision, the limits of detection (LOD) and quantification (LOQ) and trueness were assessed. Since our goal was to quantify a very low concentration of GMO, the impact of imposing the limit of blank (LOB) and pooling of reactions was assessed. The LOB was assigned for each target, and the LOB-corrected data were compared to the noncorrected data.

### 3.1. Specificity

The specificity of the assays was tested both with in silico and in vitro analyses. The interactions between the primers and probes were tested using AutoDimer [30]. No significant risk of dimers between pairs of oligonucleotides (primers/probes) was observed. The multiplex specificity of the 6-plex assay was already tested by Bogožalec Košir et al. [20], using ePCR [32], while the specificity of 6-plex assays was tested using PrimerBLAST [31] in this study. In multiplex PCR, all of the possible combinations of primers can generate an unintended PCR product, if a matching template is present by chance. There were no unintended amplicons found for any of the assays. The in vitro specificity was assessed on nontarget and target transgenic material. The nontarget material (sample F, Supplementary Table S6) contained 23 GM maize lines, and the target material (sample E, Supplementary Table S6) contained 17 GM soybean lines. No cross-reactivity was observed for 6-plex and 4-plex I assays, while one target (MON89788) was positive in 4-plex II assay (Supplementary Tables S7 and S8). Although reference materials and other control samples are the most reliable material for specificity assessment, they are only characterized in terms of the presence, and rarely absence, of specific targets, meaning that traces of other GMOs may be present but not declared. Despite this fact, the signal could only be detected for MON89788, and not for the soybean specific reference gene (*Le1*), meaning that the presence of MON89788 GM soybean line cannot be confirmed. Since there was no amplification in the negative template controls, primer dimerization could not have been the reason for the signal (additional in silico analysis showed no primer dimers). And while applying the LOB correction did decrease the number of positive partitions, it did not eliminate them entirely. Assigning a specific amplitude of florescence to each individual partition in dPCR allowed us to take a deeper look in the amplification pattern of each target in the assay. Often, false positives can be observed as the ”fog” of positive partitions rising from the negative cluster, but for which the fluorescence does not reach the same amplitude as the true positives [33,34]. This effect can be matrix-dependent [33]. It is evident that the florescence of the MON89788 target is not of the same amplitude for sample F (negative control) as it is for sample E (positive control), which clearly indicates false positive signal. Such false positive signals can be overcome by setting the threshold higher in dPCR. False positive signals do not arise solely due to cross-reactivity but can also be the results of fused droplets or contaminants such as dust particles, etc. The CrystalMiner software has an integrated quality control option where the size and shape of the droplets in the crystal can be inspected. With all the above-mentioned facts taken in consideration, the false positive signal was deemed unproblematic, and further experiments were carried on with both 6-plex and 4-plex systems.

### 3.2. Precision and Limits of Detection (LOD) and Quantification (LOQ)

To assess the precision and the LOD and LOQ of the newly developed assays, two dilution series were prepared from each of the DNA mixes, one for testing the 4-plex assays and the other for the 6-plex assay (Supplementary Tables S3 and S4). Seven dilutions were tested for the 6-plex assay, and eleven for the 4-plex assays. The precision of the method is characterized by the relative standard deviation under repeatability conditions (RSDr), which are in turn defined as ”conditions where test results are obtained with the same method, on identical test items, in the same laboratory, by the same operator, using the same equipment within short intervals of time” [35]. The LOQ is defined as the minimum copy number concentration where the sample can be reliably quantified, and the LOD as the minimum copy number concentration that can be reliably detected, i.e., all technical replicates are positive [35]. The acceptance criterion for the precision of the method states that the method is precise when RSDr ≤ 25% over the whole dynamic range. Since the LOQ is the lowest copy number concentration (≤50 copies per reaction) that is still included in the dynamic range, the LOQ and precision are tightly connected.

In order to comply with the minimum performance requirements for GMO testing, the LOD should be below 25 copies per reaction [35]. The LOD was below the 25 copy threshold for all targets in all three assays, with the exception of *Le1* and MON87796 in the 6-plex assay, where the LOD could not be determined with the used dilution series (Table 1, Table 2 and Table 3 and Supplementary Tables S9–S11). Even though the LOD was not determined for MON87769, the copy number measured for the last tested dilution (dilution 7) was six copies per reaction, indicating that the LOD is even lower and is well below 25 copies.

The LOQ was at or below 50 copies per reaction for all targets in all three assays, with the exception of CV127 (129 copies per reaction) in the 4-plex II assay (Table 1, Table 2 and Table 3 and Supplementary Tables S9–S11). It ranged from 47 copies per reaction in the case of DP305423 to as low as 12 copies per reaction for CV127, for the 6-plex assay (Supplementary Table S9) and from 43 (MON87708) to 31 (MON87701) copies per reaction for the 4-plex I (Supplementary Table S10) and from 129 (CV127) to 21 (MON87769) copies per reaction for 4-plex II (Supplementary Table S11). Apart from CV127 in the 4-plex II assay, the LOQ is compliant with the acceptance criteria. Still, to see the approximation of how the assay would behave in practice, we looked at the individual experiments. When more than one experiment is run, there is a possibility that the LOQ of one experiment is not determined at the same concertation as the other, and when the “worst case scenario” is considered, the LOQ can become higher. Between the two experiments conducted for each of the assays in this study, one showed a higher LOQ for all targets except MON87769 in the 6-plex assay (the LOQ for CV127, DP305423, *Le1*, MON87708, MON87701 rose from 12, 47, <34, 28 and 32 to 35, 89, 80, 46 and 67 copies per reaction, respectively; Supplementary Table S9). One of the two experiments also showed a higher LOQ for DP305423 (32 versus 50 copies per reaction) and MON87701 (31 versus 40 copies per reaction) in the 4-plex I assay (Supplementary Table S10). No differences were observed for 4-plex II. To eliminate the possibility that the difference occurs due to different testing days, bias of the copy number determination between the two days was calculated (Supplementary Tables S9–S11). Bias was below ± 25% for all dilutions at or above the LOQ. Although such a stringent approach towards determination of the LOQ is not a part of the Definition of Minimum Performance Requirements For Analytical Methods of GMO Testing, laboratories use it to avoid any potential errors with the quantification. Thus, in our case, the highest of the values were considered as the LOQs (Table 1, Table 2 and Table 3). However, we must take in consideration that the true LOQ is between the highest LOQ and LOD. This interval can be quite wide as is in the case of CV127 in the 4-plex II assay and DP305423 in the 4-plex assay (24–129 and 47–89 copies per reaction, respectively; Supplementary Table S9) or very narrow as in the case of MON87769 (14–21 copies per reaction) in the 4-plex II assay (Supplementary Table S11).

The comparison of the LOD and LOQ of CV127 in the 6-plex and 4-plex assays, where both are lower in the former, points to the influence different combination of targets can have in a multiplex assay. Here, a substitution of three targets with one led to the increase in the LOD and LOQ for one target (CV127) but not the others (*Le1*, MON87769), proving the necessity of a thorough assay validation.

### 3.3. Determination of GM Content and the Linearity of the Assays

For each dilution in the two dilution series, the GM% was calculated for all targets above the LOD, and the mean GM% was compared to the assigned value (Supplementary Tables S12 and S13). The RSD% between the technical replicates was <15% for all targets in the 6-plex assay and <19% for all the targets in the 4-plex I and II assays. To determine whether the measured GM% is in line with the assigned value, bias was calculated using equation 4, where *value 1* is the set value, and *value 2* is the one being evaluated. A difference <±25% was acceptable. Bias to the assigned value was between 0.23% and 15% for the 6-plex assay and between 0.74 and 24.16% for the 4-plex I and II assays. These results show that the GM% determination is possible even at a very low concentration (<50 cp/rnx).

(4) $$Bias\;\left(\%\right)=\frac{value\;1-value\;2}{value\;2}*100$$

**Figure 1:**
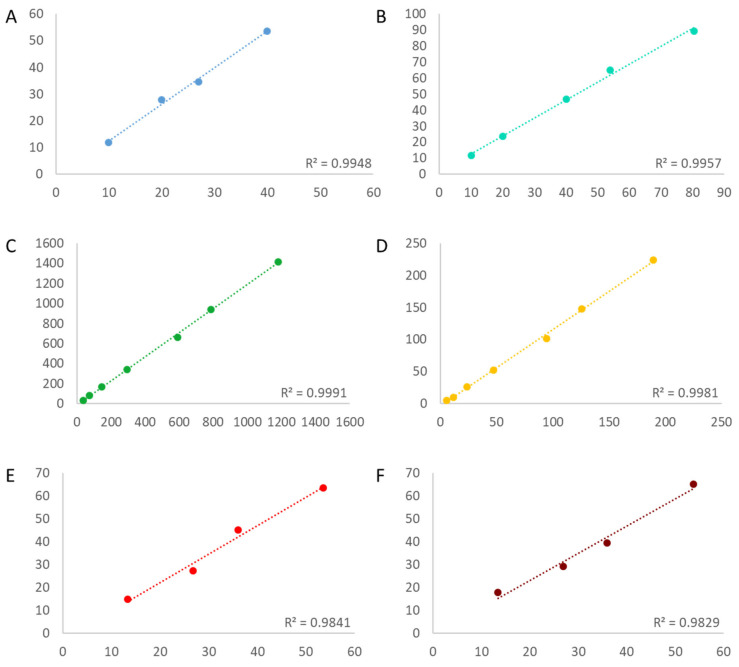
Schemes follow the same formatting. Linearity of 6-plex assay for each individual target: CV127 (**A**) blue, DP305423 (**B**) teal, *Le1* (**C**) green, MON87769 (**D**) yellow, MON87708 (**E**) red and MON87701 (**F**) infrared. R^2^ is depicted in each panel in the bottom right. X-axis = estimated copy number per reaction; Y-axis = measured copy number per reaction.

**Figure 2:**
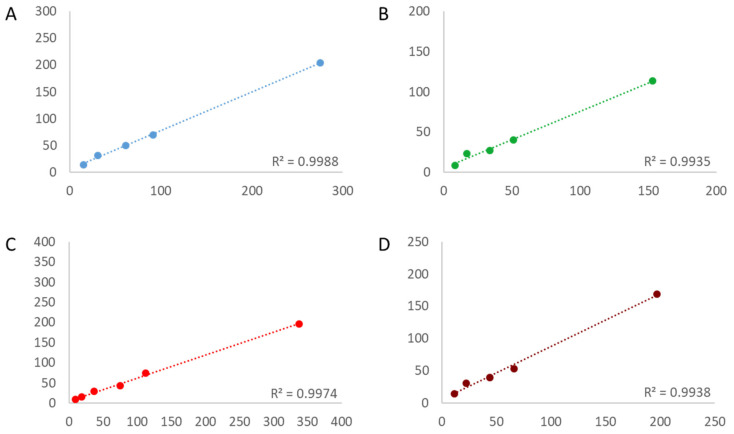
Linearity of the 4-plex I assay for each individual target: DP305423 (**A**) blue, MON40-3-2 (**B**) green, MON87708 (**C**) red, MON87701 (**D**) infrared. R^2^ is depicted in each panel in the bottom right. X-axis = estimated copy number per reaction; Y-axis = measured copy number per reaction.

**Figure 3:**
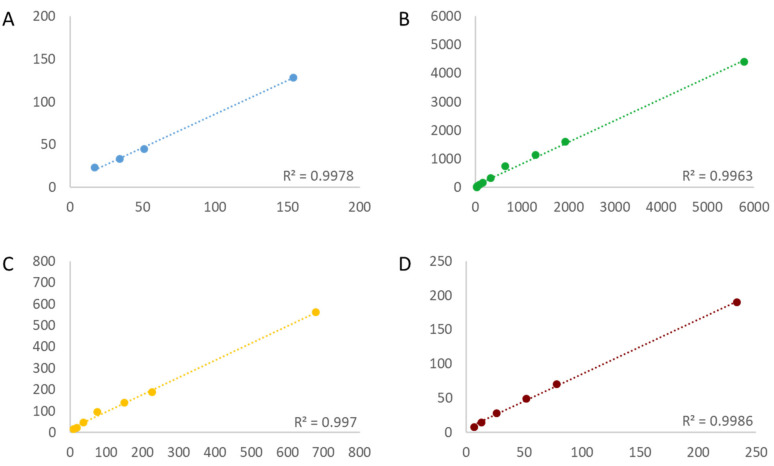
Linearity of the 4-plex II assay for each individual target: CV127 (**A**) blue, *Le1* (**B**) green, MON87769 (**C**) yellow and MON89788 (**D**) infrared. R2 is depicted in each panel in the bottom right. X-axis = estimated copy number per reaction; Y-axis = measured copy number per reaction.

In order to determine the linearity of the assays, first, the copy number of the targets needed to be estimated. The estimation was conducted by a simplex assay on the QX100 platform (BioRad), and the bias to the assigned value was calculated (Supplementary Tables S14 and S15). With R^2^ above 0.98 for all targets in all three assays, the linearity was acceptable even at a very low copy number concentration (Figure 1, Figure 2 and Figure 3). The bias between the estimated and the measured value was consistently greater for all targets in both 4-plex assays compared to the 6-plex assay.

### 3.4. Optimization of Data Analysis

Two approaches were taken in an effort to optimize the data analysis. First, to determine a more precise quantification, the limit of blank (LOB) was calculated according to a Bayesian algorithm for each target in each multiplex assay. And second, to achieve a lower LOD and higher sensitivity, the samples were pooled.

#### 3.4.1. Application of Limit of Blank (LOB)

After applying the limit-of-blank (LOB) correction, the copy number concentration for each tested dilution was lowered by as much as five cp/rnx or in other terms from 0 to 42.22% for the 6-plex, from 0.7 to 26.38% for the 4-plex I and from 0.08 to 30.69% for the 4-plex II assays (Supplementary Tables S9–S11). The LOB correction influenced both the LOQ and LOD, as it had an effect on the RSD as well as on determining the positivity of individual reactions. The limit of quantification (LOQ) decreased for all targets in the three multiplex assays, except for CV127 in the six-plex assay and MON87701 in the 4-plex II assay (Table 4 and Supplementary Tables S9–11). For these two targets, the RSD of the dilution previously considered as the LOQ was raised just above 25%, and with it the LOQ. On the other hand, a substantial LOQ reduction was also observed. For DP305423, the LOQ dropped from 89 copies per reaction to 45 copies per reaction.

With the implementation of the LOB, some individual reactions before deemed as positive were now negative; thus, it is important to note that the LOB implementation led to a reduction in sensitivity for some cases, as one of the three technical repeats was negative. The LOD interval increased for DP305423 and MON87769 in the 6-plex assay, as well as for DP305423, MON40-3-2, MON87708, *Le1* and MON89788 in the 4-plex I and II assays (Supplementary Tables S9–S11), but decreased for other targets. Nevertheless, the LOD remained below 25 copies per reaction in all instances, except for DP305423 in the 4-plex I assay, where it was just above the threshold with 29 cp/rnx. With this adjustment, a much more precise LOQ/LOD interval can be determined, which in turn facilitates a better interpretation of the results and helps to guide the decisions on further analysis if needed.

#### 3.4.2. Pooling of Reactions

When the quantitation of very low target concentrations is critical, the analyzed volume of the sample can limit the sensitivity of detection. To circumvent the limited sample volume analyzed and thereby increase analytical sensitivity, two or more chambers containing sample replicates can be pooled together and analyzed as a large single chamber. As multiple pooled chambers result in an increased total analyzed volume for a given sample (increased partition count), pooling aims to increase both the sensitivity and the precision of a given test. The implementation of pooling resulted in a two- to eightfold decrease in the LOD, down to even two copies per reaction (Supplementary Tables S9–S11).

However, it is worth noting that while pooling is highly advantageous for detecting rare mutations or infectious agents, its utility differs in the context of relative quantification of GMOs. Typically, sample abundance is not the limiting factor in the GMO quantification. In the cases where a sample exhibits an exceptionally low concentration of a specific target, it is often more cost-effective to repeat DNA extraction using a larger sample quantity or a different extraction method. Nevertheless, when dealing with nonauthorized GMOs that demand zero-tolerance presence, pooling can play a crucial role in distinguishing between the positive and negative samples, expediting legal decisions.

### 3.5. Fitness for Purpose

To assess whether the assays are fit for the quantification of low GMO content in complex samples, four real-life samples (samples A–D) containing different quantities of target GMOs were tested, while additional two samples E and F were used as the negative and positive controls, respectively. The GM content of the samples was assessed using the qPCR. A semi-quantitative approach was chosen where a difference between the Cq of *Le1* and the individual GM line is used to determine whether the GM content is below 0.1%. In short, when the difference between the Cq value of *Le1* and the individual GM line is >13 cycles, a semi-quantification can be used to determine that the GM content is below 0.1%, except for MON40-3-2 where the difference needs to exceed 12 cycles. Additionally, when the difference between the Cq values of MON40-3-2 and *Le1* is below three cycles, the sample contains more than 10% of MON40-3-2. Where this approach was not possible, the sample is marked as positive for a specific GM line, and the sample is marked as negative when no amplification was detected. The results of the analysis are summarized in Table 5 for the 6-plex assay and Table 6 for the 4-plex I and II assays. The results are comparable with those of the qPCR analysis (Supplementary Table S5), with the exception of DP305423 where the qPCR and the dPCR 4-plex I assay detected the event in samples A and B, while the event was not detected by the 6-plex assay.

While the dPCR methods have proven effective for quantifying low concentrations of GMOs in complex samples, it is essential to consider the practicality and applicability of this technology for daily use. In routine diagnostics, samples are typically not directly quantified using the qPCR. Instead, the testing process involves initial screening, the identification of specific GM lines based on the positive screening results and, finally, the quantification of the identified lines. Previous studies have demonstrated that the multiplex dPCR is more cost-effective than the single-plex qPCR when at least one sample in the analysis is positive [18,20]. In this study, we focused on six samples from the fitness-for-purpose study and compared the cost and time efficiency of the quantification with the dPCR and qPCR (Table 7).

We examined three different approaches: (1) a comparison between the direct qPCR quantification of either five or seven GM lines and the quantification using either the 6-plex assay or the two 4-plex dPCR assays, (2) a comparison between the standard screening, identification and quantification approach and the quantification with the dPCR 4-plex assays, (3) screening with the qPCR followed by the direct quantification with the dPCR. In the case of the qPCR, we considered the use of both 96- and 384-well plates, while for the dPCR, we utilized Saphire chips capable of conducting 12 25 µL reactions per experiment. In the second comparison, the premise of a positive screening test led to the identification of specific GM lines, followed by the quantification. In other words, all six samples underwent screening using a 5-plex qPCR analysis indicating the presence of specific GM elements, which suggested the presence of one or more GM lines. Based on these results, the identification of specific lines and subsequent quantification followed.

When comparing the direct quantification with the qPCR and dPCR, we observed that, regardless of the plate format used, the estimated hands-on time was shorter for the dPCR. In contrast, the direct quantification with the qPCR proved to be 2.2 to almost three times more costly than the direct quantification with the multiplex dPCR for both the 6-plex and 4-plex options. This finding is notable, as the direct quantification with the qPCR is a relatively uncommon approach, making it essential to compare it with the more standard screening/identification/quantification approach. Surprisingly, the direct quantification turned out to be slightly more cost-efficient than the standard approach, even though one sample was negative, and four samples contained four out of seven GM lines. We also explored the combination of screening using the qPCR and the direct quantification using the dPCR, which was also more cost- and time-efficient than the standard screening approach.

The quantification with the multiplex dPCR proved to be more time- and cost-efficient than the qPCR. However, this is only the case for positive samples. For negative samples, the fastest and most cost-efficient method remains the qPCR, particularly when screening with the multiplex qPCR is implemented. Taking this into consideration, a mixed approach of screening with the qPCR and the quantification using the multiplex dPCR would be the most efficient choice when the screening indicates a positive sample.

## 4. Conclusions

In the context of the rising complexity and diversity of genetically modified organisms, this study introduces a multiplex dPCR method enabling the quantification of five GM soybean lines in a single reaction. This is to our knowledge the first report of a multiplex dPCR analysis enabling the quantification of individual GM lines with such high multiplexing ability. The developed 6-plex assay complied with the recommendations for GMO testing, including the LOD and repeatability. The additional flexibility of multiplexing in the dPCR was shown by adding two GM soybean lines to the 6-plex assay and dividing it into two 4-plex assays. Although no additional optimization was performed for the 4-plex assays, they demonstrated high compliance with the recommendations. Applying the LOB correction contributed to a more accurate assessment of concentration and thus to the precision of the developed methods, while pooling increased sensitivity, proving useful in distinguishing the positive and negative signals. All the developed multiplex assays demonstrated practicability, proving to be a cost- and time-efficient approach for the quantification of low amounts of GMOs in complex samples.

## Figures and Tables

**Table 1:** LOD (bold) and LOQ (italic) of the 6-plex assay according to the total copy numbers per reaction (cp/rnx) and according to the stringent approach (LOD—bold, LOQ—underlined).

Dilution	CV127		DP305423		*Le1* ^b^		MON87769 ^c^		MON87708		MON87701	
	Mean cp/rnx	RSD%	Mean cp/rnx	RSD%	Mean cp/rnx	RSD%	Mean cp/rnx	RSD%	Mean cp/rnx	RSD%	Mean cp/rnx	RSD%
1	54	14.79	89	16.80	1418	2.59	225	9.65	64	15.08	67	16.24
2	35	22.19	65	20.39	943	5.58	149	9.45	46	21.73	40	24.54
3	*28*	*21.70*	*47*	*19.11*	660	6.76	104	18.71	*28*	*24.06*	*32*	*12.24*
4	**12**	**24.27**	24	27.42	341	7.17	53	18.77	**15**	**30.35**	**18**	**33.14**
5	7 ^a^	ND	**12**	**49.81**	167	10.46	*27*	*17.19*	9 ^a^	ND	9 ^a^	ND
6	8 ^a^	ND	8 ^a^	ND	80	10.49	11	37.90	7 ^a^	ND	Neg	ND
7	Neg	ND	6 ^a^	ND	34	21.38	6	20.88	Neg	ND	Neg	ND

^a^ At least one replicate was negative; ^b^ neither the LOD nor LOQ was reached for *Le1;* ^c^ LOD was not reached for MON87769; ND: not determined due to negative replicate(s); Neg: all replicates were negative.

**Table 2:** LOD (bold) and LOQ (italic) of the 4-plex I assay according to the total copy numbers per reaction and according to the stringent approach (LOD—bold, LOQ—underlined).

Dilution	DP305423		MON40-3-2		MON87708		MON87701	
	Mean cp/rnx	RSD%	Mean cp/rnx	RSD%	Mean cp/rnx	RSD%	Mean cp/rnx	RSD%
1	204	10.60	114	7.49	196	5.48	169	13.40
2	70	8.49	*40*	*13.68*	74	14.84	54	15.16
3	50	14.81	27	26.55	*43*	*16.43*	40	22.93
4	*32*	*23.80*	23	26.77	30	30.13	*31*	*18.36*
5	**14**	**46.11**	**9**	**42.79**	15	30.00	**14**	**35.56**
6	6 ^a^	ND	5 ^a^	ND	**9**	**31.27**	7 ^a^	ND
7	5 ^a^	ND	Neg	ND	Neg	ND	Neg	ND
8	Neg	ND	Neg	ND	Neg	ND	Neg	ND
9	Neg	ND	Neg	ND	Neg	ND	Neg	ND
10	Neg	ND	Neg	ND	Neg	ND	Neg	ND
11	Neg	ND	Neg	ND	Neg	ND	Neg	ND

^a^ At least one replicate was negative; ND: not determined due to negative replicate(s); Neg: all replicates were negative.

**Table 3:** LOD (bold) and LOQ (italic) of the 4-plex II assay according to the total copy numbers per reaction and according to the stringent approach (LOD—bold, LOQ—underlined).

Dilution	CV127		*Le1*		MON87769		MON87708	
	Mean cp/rnx	RSD%	Mean cp/rnx	RSD%	Mean cp/rnx	RSD%	Mean cp/rnx	RSD%
1	*129*	*8.60*	4417	4.63	562	4.08	190	8.84
2	45	27.98	1606	4.18	187	7.16	70	17.78
3	33	19.79	1139	4.36	139	13.85	49	12.28
4	**24**	**23.57**	740	7.57	96	18.96	*29*	*15.06*
5	11 ^a^	ND	330	8.31	48	12.53	14	28.88
6	6 ^a^	ND	177	10.67	*21*	*20.88*	**8**	**43.66**
7	4 ^a^	ND	94	9.90	**14**	**27.50**	6 ^a^	ND
8	Neg	ND	*45*	*13.71*	7 ^a^	ND	Neg	ND
9	Neg	ND	22	36.48	Neg	ND	Neg	ND
10	Neg	ND	**15**	**31.14**	Neg	ND	Neg	ND
11	Neg	ND	10 ^a^	ND	Neg	ND	Neg	ND

^a^ At least one replicate was negative; ND: not determined due to negative replicate(s); Neg: all replicates were negative.

**Table 4:** Comparison of LOQ and LOD before and after LOB correction.

Multiplex	Target	LOQ Mean cp/rnx			LOD Mean cp/rnx	
		Nonstringent	Stringent	After LOB	Before LOB	After LOB
6-plex	CV127	12	35	49	12	7
	DP305423	47	89	45	12	22
	*Le1*	NA	NA	NA	NA	NA
	MON87769	27	27	25	<6	8
	MON87708	28	46	42	15	12
	MON87701	32	67	64	18	16
4-plex I	DP305423	32	50	47	14	29
	MON40-3-2	40	40	34	9	17
	MON87708	43	43	42	9	13
	MON87701	31	40	51	14	11
4-plex II	CV127	129	129	126	24	20
	*Le1*	45	45	41	15	17
	MON87769	21	21	18	14	11
	MON89788	29	29	25	8	10

**Table 5:** 6-plex assay assessment of fitness for purpose on four real-life samples (A–D) including a positive (E) and negative (F) controls.

Event	Sample A	Sample B	Sample C	Sample D	Sample E	Sample F
	GM%	GM%	GM%	GM%	GM%	GM%
DP305423	Neg	Neg	Neg	Neg	0.78	Neg
MON87701	Neg	Neg	18.77	32.75	7.59	Neg
MON87708	0.01	0.08	23.09	1.37	6.61	Neg
CV127	Neg	Neg	Neg	Neg	2.34	Neg
MON87769	Neg	Neg	Neg	Neg	8.80	Neg

Neg: the sample was negative for the targeted GMO.

**Table 6:** 4-plex assays I and II assessment of fitness for purpose on four real-life samples (A–D) including a positive (E) and negative (F) controls.

Event	Sample A	Sample B	Sample C	Sample D	Sample E	Sample F
	GM%	GM%	GM%	GM%	GM%	GM%
DP305423	0.01	0.002	Neg	Neg	0.92	Neg
MON87701	Neg	Neg	19.26	36.45	8.12	Neg
MON87708	0.05	0.16	23.06	1.19	7.85	Neg
CV127	Neg	Neg	Neg	Neg	2.36	Neg
MON87769	Neg	Neg	Neg	Neg	8.85	Neg
MON40-3-2	0.26	0.93	25.53	35.43	4.62	Neg
MON89788	0.12	0.26	28.98	23.86	9.57	Neg

Neg: the sample was negative for the targeted GMO.

**Table 7:** Comparison of cost and time efficiency of the developed multiplex dPCR methods to the qPCR approach.

Approach	System	Rnx/Experiment ^c^	No of Experiments	Estimated Hands-On Time (h)	Relative Final Price
(1)	6-plex dPCR	12	2	3.5	1
	4-plex dPCR	12	3	4.5	1
(1)	qPCR direct quantification Five GM lines	96	5	12.5	2.96
		384	2	6.5	2.39
	qPCR direct quantification Seven GM lines	96	7	11	2.37
		384	2	8	2.20
(2)	qPCR screening ^a^ + identification + quantification ^b^	96	7	16	2.94
		384	5	13.5	2.53
(3)	qPCR screening + 6-plex dPCR	96	3	6	1.76
		384	3	6	1.14
	qPCR screening + 4-plex dPCR	96	4	7	1.57
		384	4	7	1.39

^a^ A 5-plex qPCR analysis used for the screening of the most common GM elements [36]. ^b^ The price was compared to that of the direct quantification using the 4-plex assays. ^c^ The number denotes the number of reactions for a full chip/plate.

## Data Availability

The data presented in this study are available in Supplementary Materials.

## Supplementary Materials

The following supporting information can be downloaded at https://www.mdpi.com/article/10.3390/foods12224156/s1. BogozalecKosir_FoodControl_2023_Supplement.
