# Supplementary material for: Fast and Accurate Multiplex Identification and Quantification of Seven Genetically Modified Soybean Lines Using Six-Color Digital PCR
Source: Foods. 2023 Nov 17;12(22):4156. doi: 10.3390/foods12224156 (PMC10670894; doi:10.3390/foods12224156)
Supplement: Supplementary file 1 [file foods-12-04156-s001.zip › foods-2680729-supplementary.pdf]

Supplementary material to:

Fast and Accurate Multiplex Identification and Quantification of Seven Genetically Modified Soybean Lines Using Six-Color Digital PCR

Alexandra Bogožalec Košir 1, Sabine Muller 2, Jana Žel 1, Mojca Milavec 1, Allison C. Mallory 2

and David Dobnik 1

1.Department of Biotechnology and Systems Biology, National Institute of Biology, Večna pot 121,

1000 Ljubljana, Slovenia

2. Stilla Technologies, Biopark 1, Mail du Professeur Georges Mathé, 94800 Villejuif, France

Table of contents:

Table S1: Assays used in this study. 2

Table S2: Primer and probe sequences and their final concentrations in the PCR reaction. 3

Table S3: Estimated copy number (cp/rnx) and GM % for DNA mix used in 6-plex assay testing. 4

Table S4: Estimated copy number (cp/rnx) and GM % for DNA mix used in 4-plex I and 4-plex II assay testing. 5

Table S5: Composition of real-life samples, relevant to targets in 4- and 6-plex assays, and GM% of the events. 6

Table S6: Genetically modified soybean and maize lines present in samples E and F, respectively. 7

Table S7: *In vitro* specificity assessment of the 6-plex assay. 8

Table S8: *In-vitro* specificity assessment of the 4-plex assays. 9

Table S9: 6-plex assay - limit of detection (LOD, green), limit of quantification (LOQ blue), and corresponding relative standard deviation (RSD %) for individual experiments and mean values including LOB correction and pooling. 10

Table S10: 4-plex I assay - Limit of detection (LOD, green), limit of quantification (LOQ blue), and corresponding relative standard deviation (RSD%) for individual experiments and mean values including LOB correction and pooling. 12

Table S11: 4-plex II assay - limit of detection (LOD, green), limit of quantification (LOQ blue), and corresponding relative standard deviation (RSD%) for individual experiments and mean values including LOB correction and pooling. 14

Table S12: Determination of the GM % of each target in the 6-plex assay, and the comparison to the assigned value. 16

Table S13: Determination of the GM % of each target in the 4-plex I and II assays, and the comparison to the assigned value. 17

Table S14: Bias of the measure copy number per reaction (cp/rnx) of the 6-plex assay to the estimated copy number. 18

Table S15: Bias of the measure copy number per reaction (cp/rnx) of the 4-plex I assays to the estimated copy number. 19

Table S16: Bias of the measure copy number per reaction (cp/rnx) of the 6-plex assay to the 4-plex in real-life samples. 21

Figure S1: Naica Crystal Digital PCR step-by-step 22

Figure S2: Chip layout for 6-plex assay 23

Figure S3: Chip layout for 4-plex assays I and II 24

Minimum Information for Publication of Quantitative Digital PCR Experiments for 2020" (dMIQE2020) checklist 25

Table S1: Assays used in this study.

Event/gene	OECD unique identifier	Multiplex assay[a]	Method reference[b]
DP305423	DP-3Ø5423-1	6-plex / 4-plex I	QT-EVE-GM-008
MON87701	MON-877Ø1-2	6-plex / 4-plex I	QT-EVE-GM-010
MON87708	MON-877Ø8-9	6-plex / 4-plex I	QT-EVE-GM-012
CV127	BPS-CV127-9	6-plex / 4-plex II	QT-EVE-GM-011
MON87769	MON-87769-7	6-plex / 4-plex II	QT-EVE-GM-002
Le1	/	6-plex / 4-plex II	QT-TAX-GM-002
MON40-3-2	MON-Ø4Ø32-6	4-plex I	QT-EVE-GM-005
MON89788	MON-89788-1	4-plex II	QT-EVE-GM-006

a - multiplex assay in which the event/gene is present.
b - reference of the EURL-GMFF validated method.

Table S2: Primer and probe sequences and their final concentrations in the PCR reaction.

Event/gene	Forward / reverse / probe (F/ R/P[a])	Sequence	Final concentration in PCR (nM)	Amplicon length (bp)
DP305423	F	5'-CGTGTTCTCTTTTTGGCTAGC-3'	800	93
	R	5'-GTGACCAATGAATACATAACACAAACTA-3'	500	
	P[b]	5'-**FAM/YY**-TGACACAAATGATTTTCATACAAAAGTCGAGA-BHQ-3'	220	
MON87701	F	5'-TGGTGATATGAAGATACATGCTTAGCAT-3'	600	89
	R	5'-CGTTTCCCGCCTTCAGTTTAAA-3'	600	
	P	5'-**Cy5.5**-TCAGTGTTTGACACACACACTAAGCGTGCC-BHQ-3'	250	
MON87708	F	5'-TCATACTCATTGCTGATCCATGTAG-3'	300	91
	R	5'-AGAACAAATTAACGAAAAGACAGAACG-3'	300	
	P	5'-**Cy5**-TCCCGGACTTTAGCTCAAAATGCATGTA-BHQ-3'	150	
CV127	F	5'-AACAGAAGTTTCCGTTGAGCTTTAAGAC-3'	400	88
	R	5'-CATTCGTAGCTCGGATCGTGTAC-3'	400	
	P	5'-**FAM**-TTTGGGGAAGCTGTCCCATGCCC-BHQ-3'	100	
MON87769	F	5'-CATACTCATTGCTGATCCATGTAGATT-3'	600	87
	R	5'-GCAAGTTGCTCGTGAAGTTTTG-3'	600	
	P	5'-**ROX**-CCCGGACATGAAGCCATTTACAATTGAC-BHQ -3'	200	
Le1	F	5'-CCAGCTTCGCCGCTTCCTTC-3'	650	74
	R	5'-GAAGGCAAGCCCATCTGCAAGCC-3'	650	
	P[c]	5'-**HEX/Cy3**-CTTCACCTTCTATGCCCCTGACAC-BHQ-3'	180	
MON40-3-2	F	5'-TTCATTCAAAATAAGATCATACATACAGGTT-3'	600	84
	R	5'-GGCATTTGTAGGAGCCACCTT-3'	600	
	P	5'-**HEX**-CCTTTTCCATTTGGG-MGBNFQ-3'	200	
MON89788	F	5'-TCCCGCTCTAGCGCTTCAAT-3'	1000	139
	R	5'-TCGAGCAGGACCTGCAGAA-3'	1000	
	P	5'-**Cy5.5**-CTGAAGGCGGGAAACGACAATCTG-BHQ-3'	500	

a – in the simplex assays on QX100 platform used for determination of copy number in non-blinded samples, all probes targeting GM lines were labelled with FAM and *Le1* was labelled with HEX

b – in the 4-plex I assay the probe is labelled with FAM and in the 6-plex assay with YY

c – in the 4-plex II assay the probe is labelled with HEX and in the 6-plex assay with Cy3

Table S3: Estimated copy number (cp/rnx) and GM % for DNA mix used in 6-plex assay testing.

Event / gene	GM %	Dilution 1	Dilution 2	Dilution 3	Dilution 4	Dilution 5	Dilution 6	Dilution 7
DP305423	6.82	92	61	46	23	12	6	3
MON87708	4.54	61	41	31	15	8	4	2
CV127	3.38	46	30	23	11	6	3	1
MON87769	16.04	217	144	108	54	27	14	7
MON87701	4.56	62	41	31	15	8	4	2
Le1	/	1351	900	675	338	169	84	42

Table S4: Estimated copy number (cp/rnx) and GM % for DNA mix used in 4-plex I and 4-plex II assay testing.

Event / gene	GM %	Dilution 1	Dilution 2	Dilution 3	Dilution 4	Dilution 5	Dilution 6	Dilution 7	Dilution 8	Dilution 9	Dilution 10	Dilution 11
MON40-3-2	2.65	174	58	40	20	9	5	2	1	1	0	0
MON89788	4.03	265	88	61	30	14	7	4	2	1	1	0
DP305423	4.75	312	104	71	35	17	9	4	2	1	1	0
MON87708	5.83	382	127	88	44	21	11	5	3	1	1	0
CV127	2.66	174	58	40	20	10	5	3	1	1	0	0
MON87769	11.73	770	257	176	88	42	22	11	6	3	2	1
MON87701	3.40	223	74	51	25	12	6	3	2	1	1	0
Le1	/	6561	2187	1504	747	359	185	94	48	25	15	7

Table S5: Composition of real-life samples, relevant to targets in 4- and 6-plex assays, and GM% of the events.

Event	Sample A GM %	Sample B GM %	Sample C GM %	Sample D GM %
DP305423	<0.1%[a]	<0.1%	Neg	Neg
MON87701	Neg	Neg	Pos	Pos
MON87708	<0.1%[a]	Pos	Pos	Pos
CV127	Neg	Neg	Neg	Neg
MON87769	Neg	Neg	Neg	Neg
MON40-3-2	<0.1%[a]	Pos	>10%[b]	>10%[c]
MON89788	<0.1%[a]	Pos	Pos	Pos

a – in case where the ΔCq between the *Le1* and the individual GM line is > 12 cycles for MON40-3-2 and > 13 cycles for the remaining GM lines a semi-quantification can be used to determine that the content is below 0.1%.
b – in case where the ΔCq between the *Le1* and MON40-3-2 is < 3 cycles a semi-quantification can be used to determine that the content exceeds 10%.
Pos – the sample was positive for the individual GM line, but semi-quantification was not possible.
Neg – no amplification signal was detected.

Table S6: Genetically modified soybean and maize lines present in samples E and F, respectively.

Sample E			Sample F		
**Unique Identifier**	**Common Name**	**CRM**	**Unique Identifier**	**CommonName**	**CRM**
ACS-GMØØ5-3	A2704	AOCS 0707-B10	SYN-IR162-4	MIR162	AOCS 1208-A
MON-89788-1	MON89788	AOCS 0906-B	SYN-IR6Ø4-5	MIR604	ERM-BF423d
MON-Ø4Ø32-6	MON40-3-2	ERM-BF410bp	MON-89Ø34-3	MON89034	AOCS 0906-E
DP-356Ø43-5	DP356043	ERM-BF425d	MON-88Ø17-3	MON88017	AOCS 0406-D
MON-877Ø5-6	MON87705	AOCS 0210-A	MON-8746Ø-4	MON87460	AOCS 0709-A
MON-877Ø8-9	MON87708	AOCS 0311-A	MON-ØØØ21-9	GA21	AOCS 0407-B
MST-FGØ72-2	FG72	AOCS 0610-A3	DP-Ø9814Ø-6	DP98140	ERM-BF427d
DAS-68416-4	DAS68416	ERM-BF432d	DAS-Ø15Ø7-1	DAS1507	ERM-BF418d
DP-3Ø5423-1	DP305423	ERM-BF426d	MON-ØØ6Ø3-6	NK603	ERM-BF415f
BPS-CV127-9	CV127	AOCS 0911-C	MON-ØØ863-5	MON863	ERM-BF416d
MON-877Ø1-2	MON87701	AOCS 0809-A	MON-ØØ81Ø-6	MON810	ERM-BF413gk
ACS-GMØØ6-4	A5547	AOCS 0707-C6	DAS-59122-7	DAS59122	ERM-BF424d
DAS-81419-2	DAS8141	ERM-BF437e	SYN-BT Ø11-1	Bt11	ERM-BF412f
DAS-444Ø6-6	DAS44406	ERM-BF436b	SYN-EV176-9	Bt176	ERM-BF411f
SYN-ØØØH2-5	SYTH0H2	AOCS 0809-B	SYN-E3272-5	3272	ERM-BF420c
MON-87769-7	MON87769	ND	DAS-4Ø278-9	DAS40278	ERM-BF433d
MON-87751-7	MON87751	ND	SYN-Ø53Ø7-1	5307	ND
			MON-87427-7	MON87427	AOCS 0512-A
			MON-87411-9	MON87411	ND
			VCO-Ø1981-5	1981	ERM-BF438b
			DP-ØØ4114-3	DP4114	ERM-BF439b
			MON874Ø3-1	MON87403	ND
			SYN-ØØØJG-2	MZHG0JG	ND

ND – material from other sources

Table S7: *In vitro* specificity assessment of the 6-plex assay.

Material	Target	cp / rnx			GM %
		Technical repeat 1	Technical repeat 1	Mean	
Sample F	DP305423	0	0	0	0
	MON87708	0	0	0	0
	CV127	0	0	0	0
	MON87769	0	0	0	0
	MON87701	0	0	0	0
	Le1	0	0	0	NA
Sample E	DP305423	343	375	359	0.78
	MON87708	2944	3101	3022	6.61
	CV127	1047	1094	1070	2.34
	MON87769	4048	4004	4026	8.80
	MON87701	3555	3393	3474	7.59
	Le1	46309	45197	45753	NA

Table S8: *In-vitro* specificity assessment of the 4-plex assays.

Material	Assay	Target	cp / rnx Technical repeat 1	cp / rnx Technical repeat 2	Mean	GM %
Sample F	4-plex I	DP305423	0	0	0	0
		MON40-3-2	0	0	0	0
		MON87708	0	0	0	0
		MON87701	0	0	0	0
	4-plex II	CV127	0	0	0	0
		Le1	0	0	0	0
		MON87769	0	0	0	0
		MON89788	10	16	13	ND
Sample E	4-plex I	DP305423	383	373	378	0.92
		MON40-3-2	1869	1945	1907	4.62
		MON87708	3336	3142	3239	7.85
		MON87701	3318	3388	3353	8.12
	4-plex II	CV127	1007	937	972	2.36
		Le1	41056	41499	41277	NA
		MON87769	3599	3710	3564	8.85
		MON89788	3917	3980	3948	9.57

NA – not applicable
ND – cannot be determined due to absence of *Le1* amplification

Table S9: 6-plex assay - limit of detection (LOD, green), limit of quantification (LOQ blue), and corresponding relative standard deviation (RSD %) for individual experiments and mean values including LOB correction and pooling.

Event / Gene	Dilution	Experiment 1		Experiment 2		Mean cp / rnx	RSD % all	Bias % Experiment 1 vs experiment 2	LOB correction		Bias % LOB corrected vs mean cp / rnx	Pooled	
		cp / rnx	RSD %	cp / rnx	RSD %				Mean cp / rnx	RSD % all		Mean cp / rnx	RSD % all
CV127	1	49	12.25	58	12.81	54	14.79	-15.63	49	15.02	-9.02	49	12.18
	2	41	16.02	30	15.97	35	22.19	35.81	30	26.62	-13.69	31	27.04
	3	30	26.80	26	14.09	28	21.70	13.81	23	26.50	-19.16	22	11.24
	4	11	13.65	13	30.57	12	24.27	-13.51	7	44.50	-42.22	6	26.87
	5	9*	/	7	44.48	7*	ND	ND	Neg	ND	ND	2	13.19
	6	7*	/	Neg	/	8*	ND	ND	Neg	ND	ND	0.2	141.42
	7	Neg	/	Neg	/	Neg	ND	ND	Neg	ND	ND	Neg	
DP305423	1	88	16.77	90	19.29	89	16.80	-2.09	88	17.04	-1.73	87	0.94
	2	69	27.50	62	12.41	65	20.39	10.65	64	21.03	-2.51	64	8.07
	3	44	28.49	50	6.21	47	19.11	-10.85	45	19.50	-4.21	45	9.67
	4	23	20.10	24	36.70	24	27.42	-1.03	22	29.33	-6.93	21	1.77
	5	11	27.36	12	68.82	12	49.81	-6.51	10*	ND	ND	9	10.54
	6	9*	/	7*	/	8*	ND	ND	Neg	ND	ND	4	36.48
	7	6*	/	Neg	/	6*	ND	ND	Neg	ND	ND	1*	141.42
Le1	1	1441	2.39	1396	1.79	1418	2.59	3.20	1418	2.59	0.00	1414	2.20
	2	987	2.23	907	4.44	943	5.58	8.80	938	5.76	-0.48	938	6.35
	3	665	9.59	655	3.47	660	6.76	1.45	660	6.77	0.03	657	1.26
	4	330	7.00	352	6.58	341	7.17	-6.28	341	7.17	0.00	336	5.19
	5	161	9.12	174	11.32	167	10.46	-7.46	167	10.46	0.00	162	5.67
	6	84	10.92	76	7.83	80	10.49	10.80	80	10.49	0.00	75	7.70
	7	36	13.50	31	32.69	34	21.38	14.58	34	21.38	0.00	29	11.09
MON87769	1	220	10.12	231	9.84	225	9.65	-5.09	222	9.54	-1.37	223	3.28
	2	155	9.89	145	8.70	149	9.45	7.37	146	10.07	-1.90	147	6.34
	3	103	19.17	104	21.17	104	18.71	-0.54	99	19.41	-3.91	99	0.75
	4	51	15.44	54	23.23	53	18.77	-5.94	50	18.27	-4.18	50	2.42
	5	27	22.95	27	12.72	27	17.19	0.21	25	16.87	-9.01	25	2.79
	6	10	32.05	11	46.58	11	37.90	-8.27	8	49.16	-23.92	8	28.18
	7	6	22.13	6	23.82	6	20.88	2.61	Neg	ND	ND	3	9.82

*one technical replicate negative, Neg. – more than one technical replicate negative, ND – not determined due to negative reactions

Table S9 - continuation: 6-plex assay - limit of detection (LOD, green), limit of quantification (LOQ blue), and corresponding relative standard deviation (RSD %) for individual experiments and mean values including LOB correction and pooling.

Event / Gene	Dilution	Experiment 1		Experiment 2		Mean	RSD %	Bias %	LOB correction		Bias %	Pooled	
		cp / rnx	RSD %	cp / rnx	RSD %	cp / rnx	all	Experiment 1 vs experiment 2	Mean cp / rnx	RSD % all	LOB corrected vs mean cp / rnx	Mean cp / rnx	RSD % all
MON87708	1	63	9.97	65	20.20	64	15.08	-3.95	60	14.58	-6.08	60	0.66
	2	53	18.28	39	12.27	46	21.73	34.25	42	22.86	-7.43	43	20.91
	3	24	26.98	33	9.83	28	24.06	-28.06	24	26.39	-16.14	23	26.31
	4	14	30.67	16	33.12	15	30.35	-10.84	12	37.42	-23.80	11	9.17
	5	7*	/	11	46.75	9	ND	ND	Neg	ND	ND	4	90.50
	6	7*	/	6*	/	7	ND	ND	Neg	ND	ND	1*	86.75
	7	Neg.	/	Neg.	/	Neg.	ND	ND	Neg	ND	ND	Neg	
MON87701	1	62	13.66	71	16.93	67	16.24	-12.87	64	16.54	-4.40	64	7.08
	2	41	29.22	40	23.60	40	24.54	2.07	38	25.86	-5.21	38	2.27
	3	34	2.59	30	16.79	32	12.24	12.54	27	17.84	-14.82	27	12.11
	4	18	30.70	19	40.19	18	33.14	-0.70	16	34.01	-11.78	16	3.55
	5	Neg	/	9*	/	9*	ND	ND	Neg	ND	ND	3	98.74
	6	Neg	/	Neg	/	/	ND	ND	Neg	ND	ND	Neg	
	7	Neg	/	Neg	/	/	ND	ND	Neg	ND	ND	Neg	12.18

*one technical replicate negative, Neg. – more than one technical replicate negative, ND – not determined due to negative reactions

Table S10: 4-plex I assay - Limit of detection (LOD, green), limit of quantification (LOQ blue), and corresponding relative standard deviation (RSD%) for individual experiments and mean values including LOB correction and pooling.

Event / Gene	Dilution	Experiment 1		Experiment 2		Mean cp / rnx	RSD% all	Bias % Experiment 1 vs experiment 2	LOB correction		Bias % LOB corrected vs mean cp / rnx	Pooled	
		cp / rnx	RSD%	cp / rnx	RSD%				Mean cp / rnx	RSD% all		Mean cp / rnx	RSD% all
DP305423	1	197	5.69	211	13.74	204	10.60	-6.51	201	10.56	-1.55	200	3%
	2	67	4.66	73	9.39	70	8.49	-8.47	67	8.55	-4.54	67	6%
	3	49	12.37	50	18.61	50	14.81	-3.04	47	15.73	-5.48	47	1%
	4	33	23.48	30	26.95	32	23.80	9.73	29	26.21	-9.33	29	5%
	5	16	45.47	11	42.25	14	46.11	44.32	Neg	ND	ND	10	29%
	6	5*	/	7	29.95	6*	ND	ND	Neg	ND	ND	2	81%
	7	5*	/	Neg	/	5*	ND	ND	Neg	ND	ND	0.2	141%
	8	Neg	/	Neg	/	Neg	ND	ND	Neg	ND	ND	Neg	ND
	9	Neg	/	Neg	/	Neg	ND	ND	Neg	ND	ND	Neg	ND
	10	Neg	/	Neg	/	Neg	ND	ND	Neg	ND	ND	Neg	ND
	11	Neg	/	Neg	/	Neg	ND	ND	Neg	ND	ND	Neg	ND
MON40-3-2	1	111	7.52	116	7.92	114	7.49	-4.01	107	8.01	-5.95	107	2%
	2	40	10.76	41	17.52	40	13.68	-3.29	34	16.61	-15.98	34	1%
	3	26	32.88	29	23.22	27	26.55	-10.30	21	33.12	-22.53	21	5%
	4	20	33.25	26	18.85	23	26.77	-21.01	17	37.07	-26.38	16	26%
	5	8	55.99	10	35.14	9	42.79	-15.75	Neg	ND	ND	2	30%
	6	5	24.26	5*	/	5*	ND	ND	Neg	ND	ND	Neg	ND
	7	Neg	/	Neg	/	Neg	ND	ND	Neg	ND	ND	Neg	ND
	8	Neg	/	Neg	/	Neg	ND	ND	Neg	ND	ND	Neg	ND
	9	Neg	/	Neg	/	Neg	ND	ND	Neg	ND	ND	Neg	ND
	10	Neg	/	Neg	/	Neg	ND	ND	Neg	ND	ND	Neg	ND
	11	Neg	/	Neg	/	Neg	ND	ND	Neg	ND	ND	Neg	ND
MON87708	1	200	6.01	193	4.82	196	5.48	4.01	195	5.59	-0.70	195	3%
	2	69	5.41	80	16.96	74	14.84	-14.19	73	15.02	-1.66	73	11%
	3	45	11.90	41	21.57	43	16.43	9.06	42	16.78	-2.93	41	8%
	4	28	41.06	32	21.62	30	30.13	-12.36	28	31.62	-6.00	28	11%
	5	17	26.80	13	33.55	15	30.00	24.74	13	32.54	ND	13	17%
	6	7	28.89	11	18.21	9	31.27	-35.94	7*	ND	ND	7	43%
	7	Neg	/	Neg	/	Neg	ND	-6.51	Neg	ND	ND	Neg	ND
	8	Neg	/	Neg	/	Neg	ND	-8.47	Neg	ND	ND	1*	105%
	9	Neg	/	Neg	/	Neg	ND	-3.04	Neg	ND	ND	Neg	ND
	10	Neg	/	Neg	/	Neg	ND	9.73	Neg	ND	ND	Neg	ND
	11	Neg	/	Neg	/	Neg	ND	44.32	Neg	ND	ND	Neg	ND

*one technical replicate negative, Neg. – more than one technical replicate negative, ND – not determined due to negative reactions

Table S10 - continuation: 4-plex I assay - Limit of detection (LOD, green), limit of quantification (LOQ blue), and corresponding relative standard deviation (RSD%) for individual experiments and mean values including LOB correction and pooling.

Event / Gene	Dilution	Experiment 1		Experiment 2		Mean cp / rnx	RSD% all	Bias % Experiment 1 vs experiment 2	LOB correction		Bias % LOB corrected vs mean cp / rnx	Pooled	
		cp / rnx	RSD%	cp / rnx	RSD%				Mean cp / rnx	RSD% all		Mean cp / rnx	RSD% all
MON87701	1	178	9.16	160	16.65	169	13.40	11.41	166	13.93	-1.78	167	7%
	2	55	16.17	52	15.83	54	15.16	6.10	51	16.08	-5.00	50	5%
	3	46	14.01	34	24.87	40	22.93	32.21	37	25.16	-7.59	37	19%
	4	31	11.86	30	25.58	31	18.36	2.32	28	20.40	-9.07	28	1%
	5	11	35.67	17	28.03	14	35.56	-32.50	11	47.07	-22.45	11	33%
	6	7*	/	8*	/	7*	ND	ND	4*	ND	ND	4	19%
	7	Neg	/	Neg	/	Neg	ND	ND	2	ND	ND	2	76%
	8	Neg	/	Neg	/	Neg	ND	ND	Neg	ND	ND	Neg	ND
	9	Neg	/	Neg	/	Neg	ND	ND	Neg	ND	ND	Neg	ND
	10	Neg	/	Neg	/	Neg	ND	ND	Neg	ND	ND	Neg	ND
	11	Neg	/	Neg	/	Neg	ND	ND	Neg	ND	ND	Neg	ND

*one technical replicate negative, Neg. – more than one technical replicate negative, ND – not determined due to negative reactions

Table S11: 4-plex II assay - limit of detection (LOD, green), limit of quantification (LOQ blue), and corresponding relative standard deviation (RSD%) for individual experiments and mean values including LOB correction and pooling.

Event / Gene	Dilution	Experiment 1		Experiment 2		Mean	RSD%	Bias %	LOB correction		Bias %	Pooling	
		cp / rnx	RSD%	cp / rnx	RSD%	cp / rnx	all	Experiment 1 vs experiment 2	Mean cp / rnx	RSD% all	LOB corrected vs mean cp / rnx	Mean cp / rnx	RSD% all
	1	131	4.87	126	12.01	129	8.6	3.77	126	8.74	-2.03	125	3
	2	49	28.32	42	29.08	45	27.98	17.63	42	30.52	-7.36	43	11
	3	30	15.83	36	20.66	33	19.79	-15.98	30	21.74	-9.38	30	14
	4	25	30.37	22	11.45	24	23.57	14.90	20	26.52	-15.37	20	12
	5	14*	/	9	44.49	11	ND	52.51	Neg	ND	ND	5	5
CV127	6	6*	/	6	58.13	6*	ND	ND	Neg	ND	ND	3	21
	7	5*	/	4*	/	4*	ND	ND	Neg	ND	ND	1*	71
	8	Neg	/	Neg	/	Neg	ND	ND	Neg	ND	ND	Neg	ND
	9	Neg	/	Neg	/	Neg	ND	ND	Neg	ND	ND	Neg	ND
	10	Neg	/	Neg	/	Neg	ND	ND	Neg	ND	ND	Neg	ND
	11	Neg	/	Neg	/	Neg	ND	ND	Neg	ND	ND	Neg	ND
	1	4596	1.9	4237	1.53	4417	4.63	8.47	4413	4.64	-0.08	4415	6
	2	1625	3.76	1588	4.83	1606	4.18	2.35	1603	4.18	-0.21	1600	2
	3	1169	2.85	1108	4.2	1139	4.36	5.49	1136	4.37	-0.23	1134	4
	4	785	3.1	695	5.37	740	7.57	12.86	735	7.63	-0.65	737	9
	5	345	8.01	315	6.42	330	8.31	9.37	325	8.39	-1.59	325	6
Le1	6	190	10.22	164	2.4	177	10.67	15.65	173	10.93	-2.42	173	11
	7	95	13.83	93	5.32	94	9.9	2.73	90	10.34	-4.33	89	2
	8	45	12.04	44	17.14	45	13.71	2.18	41	15.20	-8.70	40	2
	9	23	44.61	21	29.99	22	36.48	11.86	17	46.30	-21.96	17	13
	10	17	25.43	14	35.63	15	31.14	28.36	10*	ND	ND	10	28
	11	12*		9	43.57	10*	ND	ND	Neg	ND	ND	4	45
	1	580	2.28	543	2.29	562	4.08	6.75	560	4.10	-0.33	560	5
	2	183	6.31	191	8.2	187	7.16	-4.05	184	7.19	-1.78	184	3
	3	151	10.28	126	11.01	139	13.85	20.05	136	14.20	-1.99	136	13
	4	99	26.58	92	6.93	96	18.96	7.57	93	19.60	-2.94	92	5
	5	47	10.08	49	16.02	48	12.53	-2.47	44	13.64	-8.22	44	3
MON87769	6	21	24.04	21	20.92	21	20.88	0.84	18	24.61	-14.44	18	0
	7	17	23.05	12	20.45	14	27.5	40.32	11	35.60	-22.78	11	31
	8	6*	/	8*	/	7*	ND	ND	Neg	ND	ND	2	52
	9	Neg	/	Neg	/	Neg	ND	ND	Neg	ND	ND	Neg	ND
	10	Neg	/	Neg	/	Neg	ND	ND	Neg	ND	ND	Neg	ND
	11	Neg	/	Neg	/	Neg	ND	ND	Neg	ND	ND	Neg	ND

*one technical replicate negative, Neg. – all technical replicates negative, ND – not determined due to negative reactions

Table S11 - continuation: 4-plex II assay - limit of detection (LOD, green), limit of quantification (LOQ blue), and corresponding relative standard deviation (RSD%) for individual experiments and mean values including LOB correction and pooling.

Event / Gene	Dilution	Experiment 1		Experiment 2		Mean cp / rnx	RSD% all	Bias % Experiment 1 vs experiment 2	LOB correction		Bias % LOB corrected vs mean cp / rnx	Pooling	
		cp / rnx	RSD%	cp / rnx	RSD%				Mean cp / rnx	RSD% all		Mean cp / rnx	RSD% all
MON89788	1	200	9.69	180	3.31	190	8.84	10.69	187	9.02	-1.56	187	8
	2	75	19.3	66	15.08	70	17.78	14.12	67	18.61	-4.94	67	11
	3	51	13.56	47	10.13	49	12.28	9.72	46	13.06	-6.20	46	7
	4	28	16.13	29	15.9	29	15.06	-4.90	25	16.73	-12.29	25	5
	5	11	28.07	17	15.25	14	28.88	-33.89	10	46.73	-30.69	10	46
	6	10	20.59	5	27.49	8	43.66	109.92	Neg	ND	ND	4	104
	7	4*	/	7*	/	6*	ND	ND	Neg	ND	ND	1*	141
	8	Neg	/	Neg	/	Neg	ND	ND	Neg	ND	ND	Neg	ND
	9	Neg	/	Neg	/	Neg	ND	ND	Neg	ND	ND	Neg	ND
	10	Neg	/	Neg	/	Neg	ND	ND	Neg	ND	ND	Neg	ND
	11	Neg	/	Neg	/	Neg	ND	ND	Neg	ND	ND	Neg	ND

*one technical replicate negative, Neg. – all technical replicates negative, ND – not determined due to negative reactions

Table S12: Determination of the GM % of each target in the 6-plex assay, and the comparison to the assigned value.

Event	Experiment	Estimated GM %	Dilution							Mean GM %	RSD% GM %	Bias to estimated GM %
			1	2	3	4	5	6	7			
CV127	1	3.38	3.41	4.16	4.46	3.41	ND	ND	ND	3.88	11.02	14.80
	2		4.17	3.34	3.98	3.69	4.31	ND	ND			
DP305423	1	6.82	6.13	6.99	6.67	7.08	6.97	ND	ND	6.84	5.69	0.23
	2		6.46	6.87	7.59	6.71	6.90	ND	ND			
MON87769	1	16.04	15.24	15.73	15.53	15.46	17.00	12.33	16.72	15.79	8.74	-1.57
	2		16.58	15.94	15.84	15.41	15.69	14.89	18.68			
MON87708	1	4.54	4.37	5.37	3.55	4.39	ND	ND	ND	4.70	14.67	3.56
	2		4.69	4.35	5.01	4.61	5.97	ND	ND			
MON87701	1	4.56	4.30	4.13	5.12	5.58	ND	ND	ND	4.81	10.85	5.59
	2		5.09	4.40	4.62	5.27	ND	ND	ND			

ND – GM% not determined dilution <LOD

Table S13: Determination of the GM % of each target in the 4-plex I and II assays, and the comparison to the assigned value.

Event	Experiment	Assigned GM %	Dilution											Mean GM %	RSD% GM %	Bias to assigned %
			1	2	3	4	5	6	7	8	9	10	11			
CV127	1	2.66	2.85	3.02	2.59	3.22	ND	ND	ND	ND	ND	ND	ND	3.03	10.62	13.83
	2		2.98	2.62	3.25	3.16	2.90	3.67	ND	ND	ND	ND	ND			
DP305423	1	4.75	4.29	4.13	4.19	4.27	4.77	ND	ND	ND	ND	ND	ND	4.38	8.70	-7.81
	2		4.98	4.62	4.56	4.39	3.61	ND	ND	ND	ND	ND	ND			
MON87769	1	11.73	12.62	11.29	12.95	12.66	13.72	11.11	17.45	ND	ND	ND	ND	13.02	12.87	10.98
	2		12.83	12.04	11.38	13.28	15.39	12.74	12.77	ND	ND	ND	ND			
MON87708	1	5.83	4.36	4.22	3.86	3.55	4.83	3.61	ND	ND	ND	ND	ND	4.42	18.34	-24.16
	2		4.54	5.03	3.74	4.57	4.23	6.51	ND	ND	ND	ND	ND			
MON87701	1	3.40	3.88	3.40	3.90	3.97	3.31	3.61	ND	ND	ND	ND	ND	3.82	16.60	12.27
	2		3.77	3.28	3.11	4.38	5.37	ND	ND	ND	ND	ND	ND			
MON40-3-2	1	2.65	2.42	2.45	2.19	2.60	2.34	ND	ND	ND	ND	ND	ND	2.67	16.25	0.74
	2		2.74	2.59	2.58	3.71	3.04	ND	ND	ND	ND	ND	ND			
MON89788	1	4.03	4.34	4.62	4.39	3.54	3.33	5.49	ND	ND	ND	ND	ND	4.26	17.57	5.56
	2		4.26	4.15	4.22	4.20	5.51	3.03	ND	ND	ND	ND	ND			

ND – GM% not determined dilution <LOD

Table S14: Bias of the measure copy number per reaction (cp/rnx) of the 6-plex assay to the estimated copy number.

Event	Dilution	**Estimated value** cp/rnx	**Measured** cp/rnx	**Bias to estimated** cp/rnx
CV127	1	46	54	17.53
	2	30	35	15.21
	3	23	28	22.02
	4	11	12	6.09
	5	6	< LOD	ND
	6	3	< LOD	ND
	7	1	< LOD	ND
DP305423	1	92	89	-3.13
	2	61	65	6.36
	3	46	47	2.10
	4	23	24	2.33
	5	12	12	0.82
	6	6	< LOD	ND
	7	3	< LOD	ND
Le1	1	1351	1418	5.00
	2	900	943	4.68
	3	675	660	-2.27
	4	338	341	0.94
	5	169	167	-0.88
	6	84	80	-5.14
	7	42	34	-20.38
MON89769	1	217	225	4.08
	2	144	149	3.38
	3	108	104	-4.42
	4	54	53	-2.87
	5	27	27	0.86
	6	14	11	-19.92
	7	7	6	-13.15
MON87708	1	61	64	4.65
	2	41	46	11.27
	3	31	28	-8.01
	4	15	15	0.06
	5	8	< LOD	ND
	6	4	< LOD	ND
	7	2	< LOD	ND
MON87701	1	62	67	7.94
	2	41	40	-1.94
	3	31	32	4.39
	4	15	18	19.89
	5	8	< LOD	ND
	6	4	< LOD	ND
	7	2	< LOD	ND

ND – not determined < LOD

Table S15: Bias of the measure copy number per reaction (cp/rnx) of the 4-plex I assays to the estimated copy number.

Event	Dilution	Estimated value cp/rnx	Measured cp/rnx	Bias to estimated cp/rnx
DP305423	1	312	204	-34.50
	2	104	70	-32.45
	3	71	50	-30.42
	4	35	32	-9.90
	5	17	14	-18.27
	6	9	< LOD	ND
	7	4	< LOD	ND
	8	2	< LOD	ND
	9	1	< LOD	ND
	10	0.7	< LOD	ND
	11	0.3	< LOD	ND
MON40-3-2	1	174	114	-34.48
	2	58	40	-30.09
	3	40	27	-31.91
	4	20	23	16.76
	5	9	9	-7.01
	6	5	< LOD	ND
	7	2	< LOD	ND
	8	1	< LOD	ND
	9	0.7	< LOD	ND
	10	0.4	< LOD	ND
	11	0.2	< LOD	ND
MON87708	1	382	196	-48.64
	2	127	74	-41.76
	3	88	43	-50.64
	4	44	30	-31.60
	5	21	15	-28.18
	6	11	9	-18.49
	7	5	< LOD	ND
	8	3	< LOD	ND
	9	2	< LOD	ND
	10	0.9	< LOD	ND
	11	0.4	< LOD	ND
MON87701	1	223	169	-24.23
	2	74	54	-27.79
	3	51	40	-21.70
	4	25	31	21.22
	5	12	14	16.39
	6	6	< LOD	ND
	7	3	< LOD	ND
	8	2	< LOD	ND
	9	0.8	< LOD	ND
	10	0.5	< LOD	ND
	11	0.2	< LOD	ND

ND – not determined < LOD

Table S15 - continuation: Bias of the measure copy number per reaction (cp/rnx) of the 4-plex II assays to the estimated copy number.

Event	Dilution	Estimated value cp/rnx	Measured cp/rnx	Bias to estimated cp/rnx
CV127	1	174	129	-26.28
	2	58	45	-22.03
	3	40	33	-17.23
	4	20	24	18.95
	5	10	< LOD	ND
	6	5	< LOD	ND
	7	2	< LOD	ND
	8	1	< LOD	ND
	9	0.7	< LOD	ND
	10	0.4	< LOD	ND
	11	0.2	< LOD	ND
Le1	1	6846	4417	-32.69
	2	2282	1606	-26.56
	3	1521	1139	-24.31
	4	761	740	-1.00
	5	380	330	-7.88
	6	190	177	-4.08
	7	95	94	-0.26
	8	48	45	-6.24
	9	24	22	-12.81
	10	16	15	0.38
	11	11	< LOD	ND
MON87769	1	770	562	-27.00
	2	257	187	-26.97
	3	176	139	-21.36
	4	88	96	9.32
	5	42	48	14.00
	6	22	21	-2.96
	7	11	14	28.76
	8	6	< LOD	ND
	9	3	< LOD	ND
	10	2	< LOD	ND
	11	0.8	< LOD	ND
MON89788	1	265	190	-28.20
	2	88	70	-20.08
	3	61	49	-19.16
	4	30	29	-5.40
	5	14	14	-0.19
	6	7	8	3.47
	7	4	< LOD	ND
	8	2	< LOD	ND
	9	1	< LOD	ND
	10	0.6	< LOD	ND
	11	0.3	< LOD	ND

ND – not determined < LOD

Table S16: Bias of the measure copy number per reaction (cp/rnx) of the 6-plex assay to the 4-plex in real-life samples.

Event	Sample A GM%			Sample B GM%			Sample C GM%			Sample D GM%		
	6-plex	4-plex I/II	Bias %	6-plex	4-plex I/II	Bias %	6-plex	4-plex I/II	Bias %	6-plex	4-plex I/II	Bias %
DP305423	Neg	0.01	NA	Neg	0.002	NA	Neg	Neg	/	Neg	Neg	
MON87701	Neg	Neg	/	Neg	Neg	/	18.77	19.26	2.61	32.75	36.45	11.30
MON87708	0.005	0.054	983.8	0.08	0.16	113.3	23.09	23.06	-0.13	1.37	1.19	-13.14
CV127	Neg	Neg	/	Neg	Neg	/	Neg	Neg	/	Neg	Neg	/
MON87769	Neg	Neg	/	Neg	Neg	/	Neg	Neg	/	Neg	Neg	/
MON40-3-2	NA	0.26	NA	NA	0.93	NA	NA	25.53	NA	NA	35.43	NA
MON89788	NA	0.12	NA	NA	0.26	NA	NA	28.98	NA	NA	23.86	NA

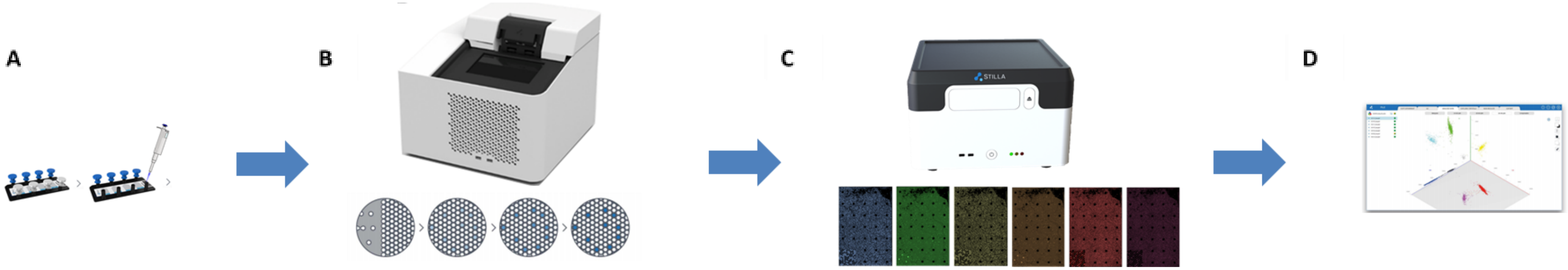

Figure S1: Naica Crystal Digital PCR step-by-step**:** pipette reaction mixture in the consumable Sapphire chip (A), transfer the chips to the Naica Geode for generation of droplet partitions followed by PCR cycling (B), after cycling transfer the chips to the Naica prototype 6-color reader for imaging (C) and analyse the readout using the prototype 6-color Crystal Miner software (D).

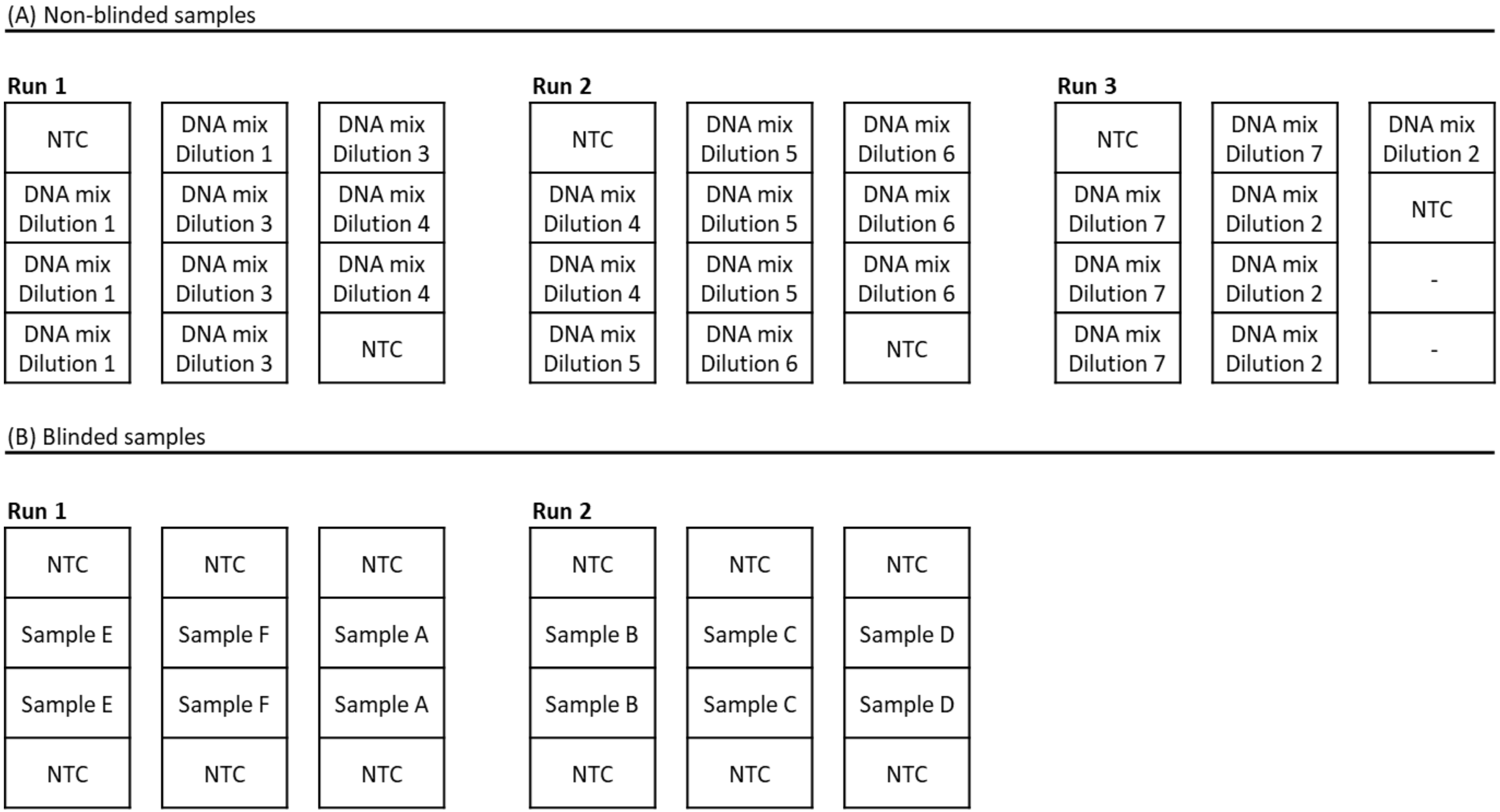

Figure S2: Chip layout for 6-plex assay. (A) non-blinded samples; the setup was repeated twice, (B) blinded samples

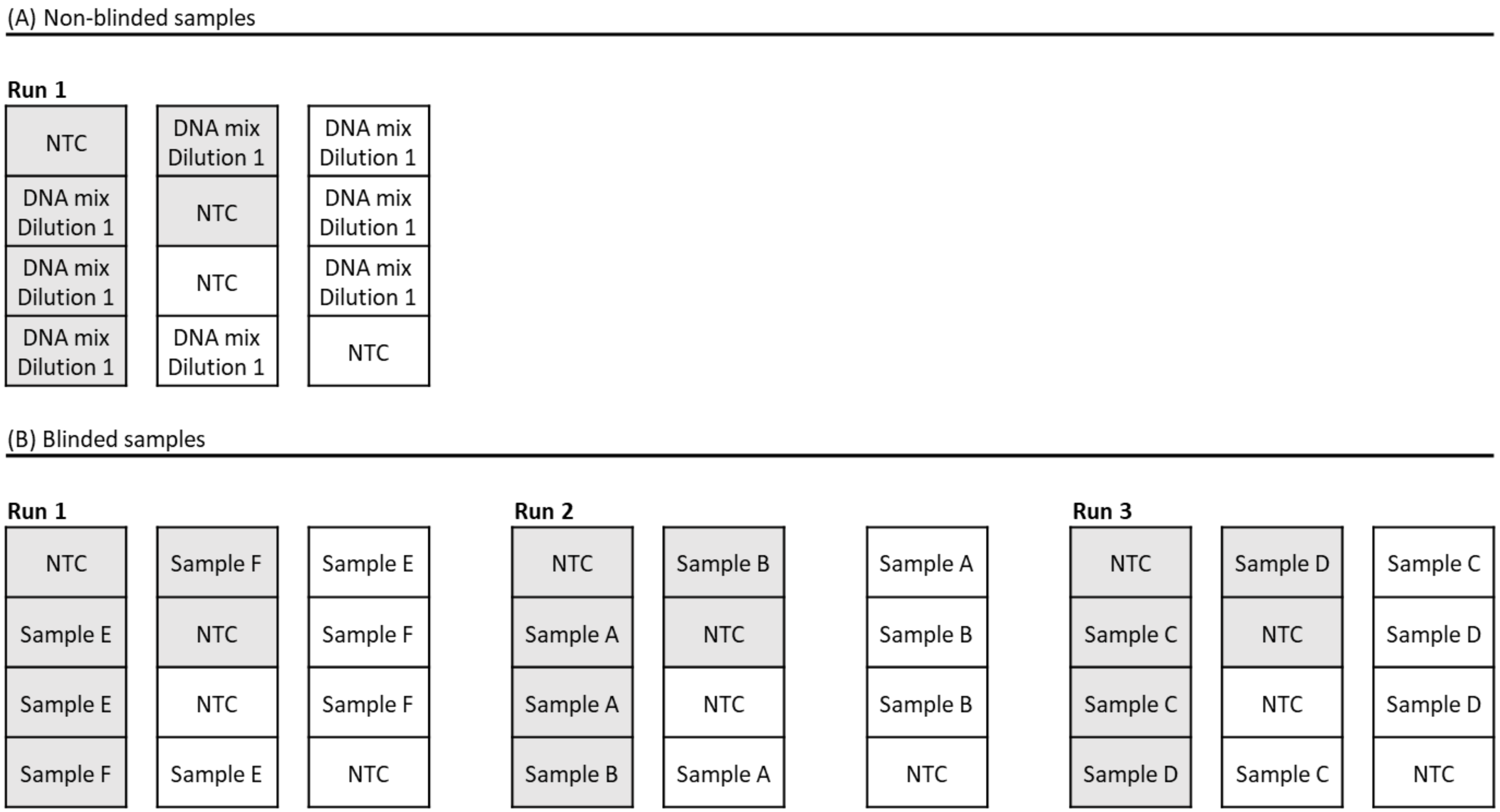

Figure S3: Chip layout for 4-plex assays I and II. (A) non-blinded samples; the setup was repeated twice for each of the 11 dilutions, (B) blinded samples. Grey cells indicate reactions on 4-plex I and white cells on 4-plex II assay.

Minimum Information for Publication of Quantitative Digital PCR Experiments for 2020" (dMIQE2020) checklist

Item to check	Provided	Comment
**1. SPECIMEN**		
Detailed description of specimen type and numbers	Y	Materials and methods (2.1 Test materials)
Sampling procedure (including time to storage)	N	
Sample aliquoting, storage conditions and duration	Y	Materials and methods (2.2 DNA extraction and purification)
**2. NUCLEIC ACID EXTRACTION**		
Description of extraction method including amount of sample processed	Y	Materials and methods (2.2 DNA extraction and purification)
Volume of solvent used to elute/resuspend extract	Y	Materials and methods (2.2 DNA extraction and purification)
Number of extraction replicates	Y	Materials and methods (2.2 DNA extraction and purification)
Extraction blanks included?	Y	Materials and methods (2.2 DNA extraction and purification)
**3. NUCLEIC ACID ASSESSMENT AND STORAGE**		
Method to evaluate quality of nucleic acids	N	
Method to evaluate quantity of nucleic acids (including molecular weight and calculations when using mass)	N	
Storage conditions: temperature, concentration, duration, buffer, aliquots	Y	Materials and methods (2.2 DNA extraction and purification)
Clear description of dilution steps used to prepare working DNA solution	Y	Materials and methods and Supplementary Material
**4. NUCLEIC ACID MODIFICATION**	**NA**	
Template modification (digestion, sonication, pre-amplification, bisulphite, etc.)		
Details of repurification following modification, if performed		
**5. REVERSE TRANSCRIPTION**	**NA**	
cDNA priming method and concentration		
One or two step protocol (include reaction details for two step)		
Amount of RNA added per reaction		
Detailed reaction components and conditions		
Estimated copies measured with and without addition of reverse transcriptase		
Manufacturer of reagents used, with catalogue and lot numbers		

Storage of cDNA: temperature, concentration, duration, buffer and aliquots		
**6. dPCR OLIGONUCLEOTIDES DESIGN AND TARGET INFORMATION**		
Sequence accession number or official gene symbol	NA	
Method (software) used for design and *in-silico* verification	Y	Materials and methods (2.3 In-silico specificity prediction)
Location of amplicon	NA	
Amplicon length	Y	Supplementary Table S6
Primer and probe sequences (or amplicon context sequence)	Y	Supplementary Table S6
Location and identity of any modifications	Y	Supplementary Table S6
Manufacturer of oligonucleotides	Y	Materials and methods (2.4 Primers, probes and PCR methods)
**7. dPCR PROTOCOL**		
Manufacturer of dPCR instrument and instrument model	Y	Materials and methods (2.5 Multiplex Crystal™ digital PCR conditions and imaging)
Buffer/kit manufacturer with catalogue and lot number	Y	Materials and methods (2.5 Multiplex Crystal™ digital PCR conditions and imaging)
Primer and probe concentrations	Y	Supplementary Table S6
Pre-reaction volume and composition (including amount of template and if restriction enzyme added)	Y	Materials and methods (2.5 Multiplex Crystal™ digital PCR conditions and imaging)
Template treatment (initial heating or chemical denaturation)	N	No template treatment prior to PCR
Polymerase identity and concentration, Mg^{++} and dNTP concentrations	N	Not disclosed by manufacturer
Complete thermocycling parameters	Y	Materials and methods (2.5 Multiplex Crystal™ digital PCR conditions and imaging)
**8. ASSAY VALIDATION**		
Details of optimisation performed	N	All assays were validated by EURL thus a validation of simplex assays was not repeated in this study.
Analytical specificity (vs. related sequences) and limit of blank	N	
Analytical sensitivity/limit of detection, and how this was evaluated	N	
Testing for inhibitors (from biological matrix/extraction)	N	
**9. DATA ANALYSIS**		
Description of dPCR experimental design	Y	Materials and methods
Comprehensive details of negative and positive controls (whether applied for quality control or for estimation of error)	Y	Materials and methods
Partition classification method (thresholding)	Y	Materials and methods
Examples of positive and negative experimental results (including fluorescence plots in Supplemental Materials)	N	
Description of technical replication	Y	Materials and methods

Repeatability (intra-experiment variation)	Y	Materials and methods
Reproducibility (inter-experiment/user/lab, etc. variation)	Y	Materials and methods
Number of partitions measured (average and standard deviation)	N	
Partition volume	N	
Copies per partition (λ or equivalent) (average and standard deviation)	N	
dPCR analysis program (source, version)	Y	Materials and methods
Description of normalisation method	N	No normalisation used
Statistical methods used for analysis	Y	Materials and methods
Data transparency	Raw data available on request	
